# Supplementary material for: Divide et impera: An In Silico Screening Targeting HCMV ppUL44 Processivity Factor Homodimerization Identifies Small Molecules Inhibiting Viral Replication
Source: Viruses. 2021 May 20;13(5):941. doi: 10.3390/v13050941 (PMC8160850; doi:10.3390/v13050941)
Supplement: Supplementary file 1 [file viruses-13-00941-s001.zip › viruses-1233575-supplementary.pdf]

Supplementary Material

Divide et impera: an *in silico* screening targeting HCMV ppUL44 processivity factor homodimerization identifies small molecules inhibiting viral replication

Hanieh Ghassabian[1], Federico Falchi[2], Martina Timmoneri[1], Beatrice Mercorelli[1], Arianna Loregian[1], Giorgio Palù[1], Gualtiero Alvisi[1*]

[1] Department of Molecular Medicine, University of Padua, Italy; haniehghassabian@gmail.com; martina.timmoneri@gmail.com; beatrice.mercorelli@unipd.it; arianna.loregian@unipd.it; Giorgio.palu@unipd.it; gualtiero.alvisi@unipd.it

[2] Molecular Horizon, Bettona (PG), Italy federico.falchi@hotmail.com

* Correspondence: gualtiero.alvisi@unipd.it;

Supplementary Figure S1

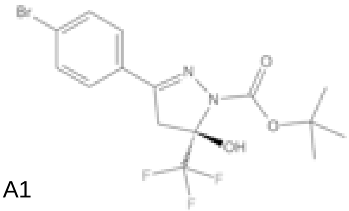
A1

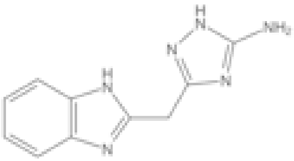
A2

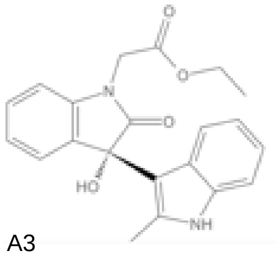
A3

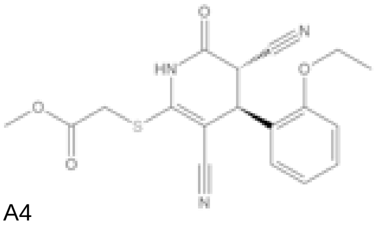
A4

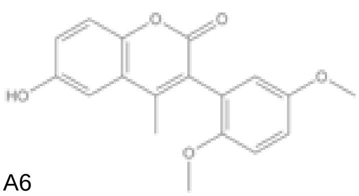
A6

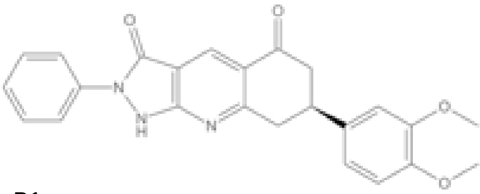
B1

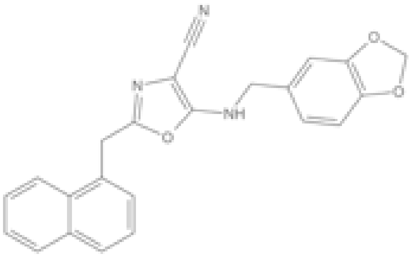
B2

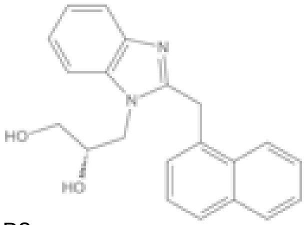
B3

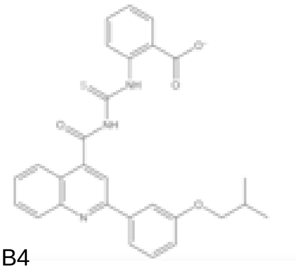
B4

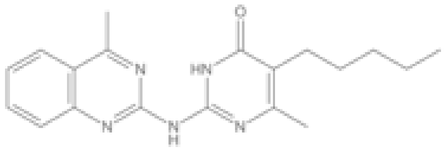
B5

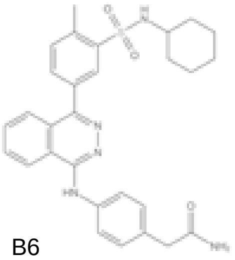
B6

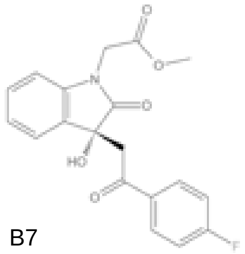
B7

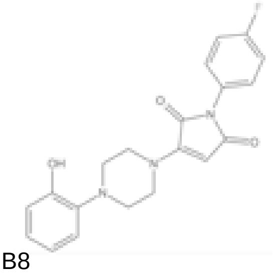
B8

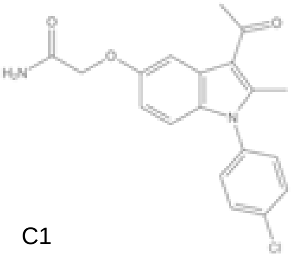
C1

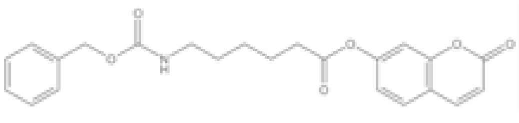
C2

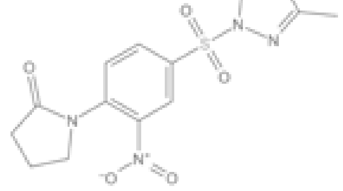
C3

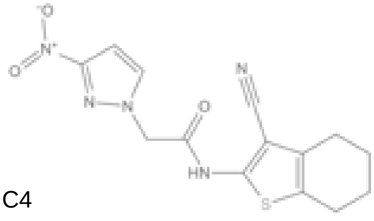
C4

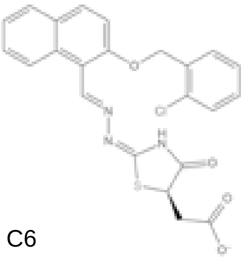
C6

Supplementary Figure S2

TB4-UL83-YFP

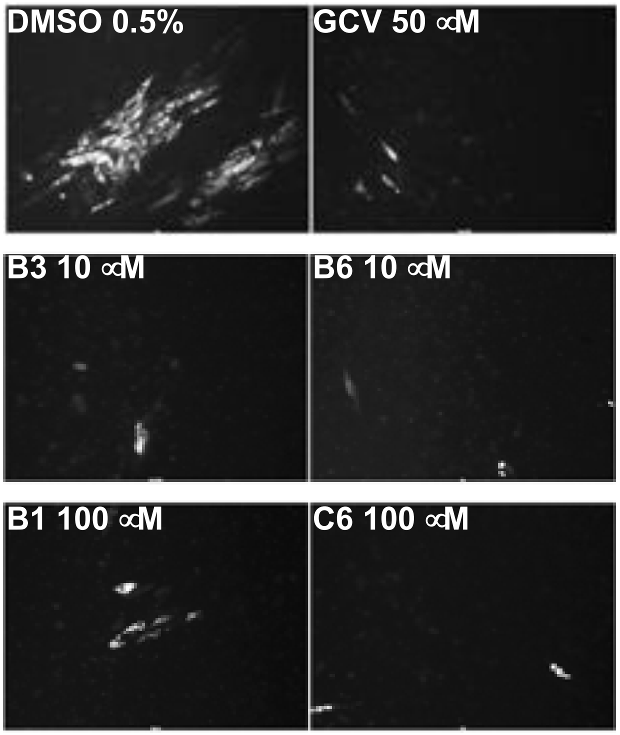

Supplementary Figure S3

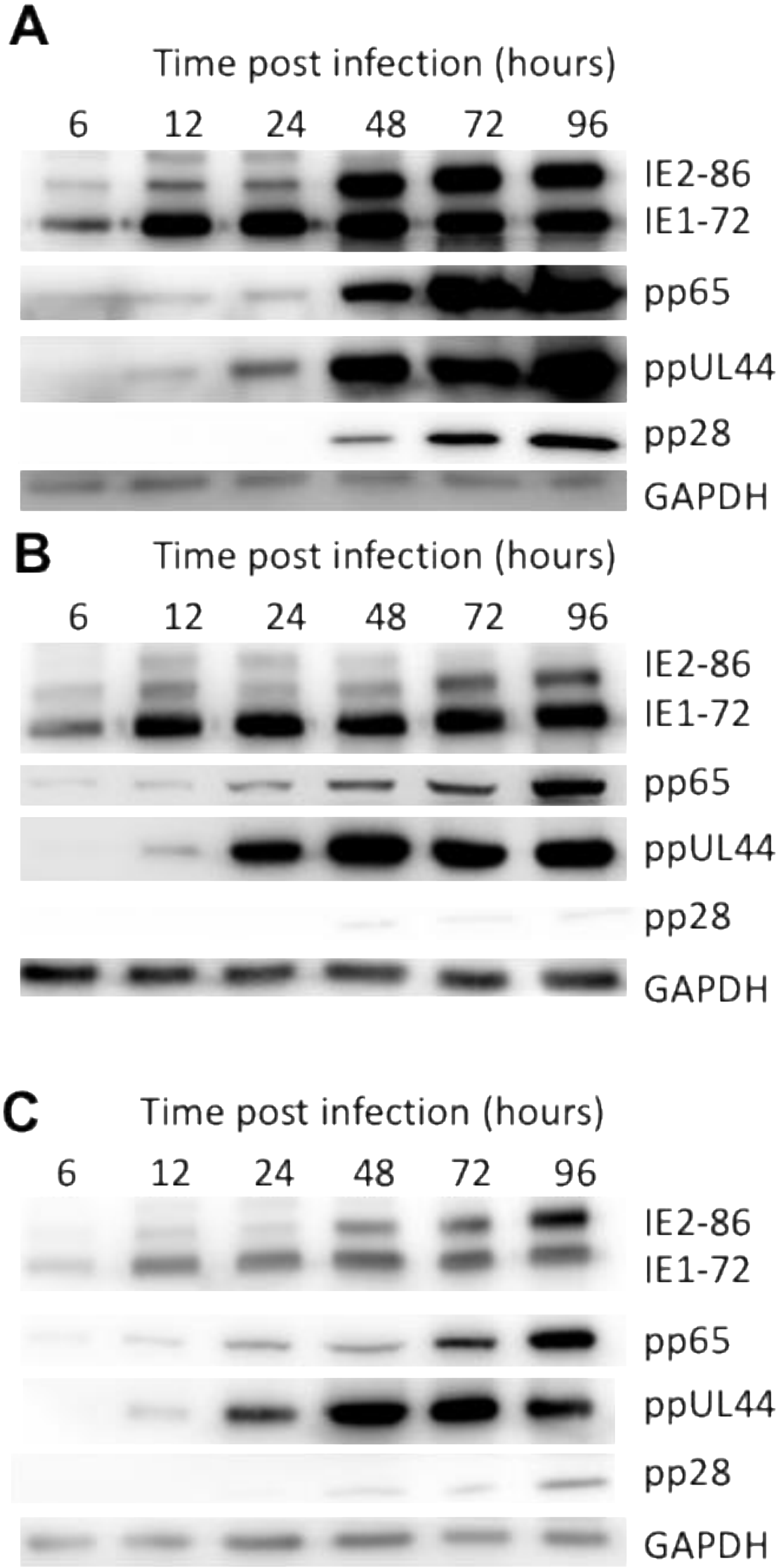
A
Time post infection (hours)
6 12 24 48 72 96
IE2-86
IE1-72
pp65
ppUL44
pp28
GAPDH
B
Time post infection (hours)
6 12 24 48 72 96
IE2-86
IE1-72
pp65
ppUL44
pp28
GAPDH
C
Time post infection (hours)
6 12 24 48 72 96
IE2-86
IE1-72
pp65
ppUL44
pp28
GAPDH

Supplementary Figure S1. Molecular structures of purchased SMs. The chemical structure of each purchased SM is shown.

Supplementary Figure S2. Treatment with selected compounds inhibits the replication of TB4-UL83-YFP reporter virus. MRC5 cells infected and treated as described in legend of Figure 2 were analyzed with a fluorescence inverted microscope, equipped with a 20x objective, a digital camera and appropriate filter set to visualize expression of YFP-UL83 at 7 days p.i.. Representative images are shown relative to cells treated as indicated.

Supplementary Figure S3. B3 specifically impairs early and late HCMV AD169 gene expression. MRC5 were infected with HCMV AD169 and treated as described in the Materials and Methods section with either vehicle alone (A, DMSO 0.5%), GCV (B, 50 μM in DMSO 0.5%) or B3 (C, 50 μM in DMSO 0.5%). At the indicated time points p.i., cells were lysed and processed for Western Blotting to detect the expression of the immediate early IE1/2 antigens, the early-late antigens ppUL44 and pp65 as well as the late antigen pp28. GAPDH was also detected as loading control.
